# Targeting the Endothelin-1 Receptors Curtails Tumor Growth and Angiogenesis in Multiple Myeloma

**DOI:** 10.3389/fonc.2020.600025

**Published:** 2021-01-08

**Authors:** Anna Russignan, Giada Dal Collo, Anna Bagnato, Nicola Tamassia, Mattia Bugatti, Mirella Belleri, Luisa Lorenzi, Enrica Borsi, Riccardo Bazzoni, Michele Gottardi, Carolina Terragna, William Vermi, Arianna Giacomini, Marco Presta, Marco Antonio Cassatella, Mauro Krampera, Cristina Tecchio

**Affiliations:** ^1^ Section of Hematology and Bone-Marrow Transplant Unit, Department of Medicine, University of Verona, Verona, Italy; ^2^ Preclinical Models and New Therapeutic Agents Unit, IRCCS Regina Elena National Cancer Institute, Rome, Italy; ^3^ Section of General Pathology, Department of Medicine, University of Verona, Verona, Italy; ^4^ Section of Pathology, Department of Molecular and Translational Medicine, University of Brescia, Brescia, Italy; ^5^ Experimental Oncology and Immunology Unit, Department of Molecular and Translational Medicine, University of Brescia, Brescia, Italy; ^6^ Department of Experimental Diagnostic and Specialty Medicine (DIMES), “L. and A. Seràgnoli”, Bologna University, Bologna, Italy; ^7^ Hematology Section, Ospedale Cà Foncello, Treviso, Italy

**Keywords:** multiple myeloma, endothelin-1 axis, macitentan, HIF-1α, angiogenesis

## Abstract

The endothelin-1 (ET-1) receptors were recently found to mediate pro-survival functions in multiple myeloma (MM) cells in response to autocrine ET-1. This study investigated the effectiveness of macitentan, a dual ET-1 receptor antagonist, in MM treatment, and the mechanisms underlying its activities. Macitentan affected significantly MM cell (RPMI-8226, U266, KMS-12-PE) survival and pro-angiogenic cytokine release by down-modulating ET-1-activated MAPK/ERK and HIF-1α pathways, respectively. HIF-1α silencing abrogated the ET-1 mediated induction of genes encoding for pro-angiogenic cytokines such as VEGF-A, IL-8, Adrenomedullin, and ET-1 itself. Upon exposure to macitentan, MM cells cultured in the presence of the hypoxia-mimetic agent CoCl_2_, exogenous ET-1, or CoCl_2_ plus ET-1, down-regulated HIF-1α and the transcription and release of downstream pro-angiogenic cytokines. Consistently, macitentan limited significantly the basal pro-angiogenic activity of RPMI-8226 cells in chorioallantoic membrane assay. In xenograft mouse models, established by injecting NOG mice either *via* intra-caudal vein with U266 or subcutaneously with RPMI-8226 cells, macitentan reduced effectively the number of MM cells infiltrating bone marrow, and the size and microvascular density of subcutaneous MM tumors. ET-1 receptors targeting by macitentan represents an effective anti-proliferative and anti-angiogenic therapeutic approach in preclinical settings of MM.

## Introduction

Multiple myeloma (MM) is a B cell malignancy arising from post-germinative B cells or plasma cell (PC) precursors and characterized by the accumulation of malignant PCs in the bone marrow (BM), the onset of monoclonal gammopathy, and eventually a significant morbidity due to organ dysfunction (1, 2). In spite of recent developments in novel therapies, MM remains an incurable disease, accounting for about 10% of all hematologic malignancies (3). Although various genomic aberrations have been shown to provide PCs with the ability to proliferate in an uncontrolled manner, increasing evidence suggests critical roles for surface receptors with restricted expression in malignant PCs and for BM microenvironment in mediating MM survival, proliferation and resistance to therapy (4).

Endothelin-1 (ET-1), originally isolated from endothelial cells (ECs), as well as its receptors A (ET_A_R) and B (ET_B_R), referred to as the “ET-1 axis”, exert key physiological functions in the human cardiovascular system and in other normal tissues (5, 6). In the last two decades the ET-1 axis has been also implicated in the development of an increasing number of tumors *via* autocrine and/or paracrine activation of pathways involved in cell proliferation, migration, invasion, epithelial-mesenchymal transition, osteogenesis and angiogenesis (7).

We have previously demonstrated that the ET-1 axis supports MM cell viability through autocrine activation, also anticipating a possible paracrine triggering (8). Accordingly, we found that both primary malignant PCs and MM cell lines were constitutively ET_A_R positive, expressing ET_B_R in roughly half of cases on an epigenetic dysregulation basis. MM cells and BM microenvironment, in particular ECs, were also found to express and release ET-1 (8). Importantly, *in vitro* experiments consisting in ET_A_R or ET_B_R blockade with selective antagonists or with the dual receptor antagonist bosentan (5) resulted in a significantly decreased viability of MM cell lines (8). More recently, preliminary *in vitro* results have demonstrated that the dual receptor antagonist macitentan also decreases MM cell viability (9). Interestingly, both bosentan and macitentan are orally active drugs already licensed in clinics for the treatment of pulmonary arterial hypertension (PAH) (10). Macitentan, designed to improve the efficacy and tolerability of bosentan, presents as main pharmacokinetic advantage a longer and sustained ET_A_R and ET_B_R occupancy that allows both an effective drug activity in the presence of elevated ET-1 levels and a one-daily dosing (10). At present, the mechanisms underlying the inhibitory activity of ET-1 receptor (ET-1R) antagonists remain to be established in MM. In this tumor context, angiogenesis is a constant hallmark of BM microenvironment during MM progression (11). Several studies have demonstrated that the hypoxia inducible factor-1alpha (HIF-1α) stabilization, favored by the hypoxic MM BM niche, induces the transcription of a few pro-angiogenic factors, including Vascular Endothelial Growth Factor A (VEGF-A), both in tumor and stromal cells (11). In solid tumors HIF-1α may be controlled also in a hypoxia-independent manner by a number of molecules, including ET-1 (12–15). Importantly, ET-1 stimulates hypoxic pathways promoting further ET-1 expression, thus maintaining angiogenic and pro-tumoral responses in a positive feedback system (16). Despite the available evidence demonstrating that HIF-1α may be regulated in a hypoxia-independent fashion even in MM (17), the regulatory effects of ET-1 on HIF-1α expression and related pro-angiogenic activities in MM is currently unknown. However, a recent network-based analysis of BM samples from patients affected by monoclonal gammopathy of undetermined significance (MGUS) and MM has highlighted the significant enrichment of ET-1 in the HIF-1α signaling pathway of primary MGUS and MM PCs (18).

Based on the aforementioned premises, aims of our work were: i) to evaluate *in vitro* the mechanisms underlying the anti-proliferative and pro-apoptotic effects exerted by macitentan in MM cells, ii) to establish *in vitro* the eventual anti-angiogenic role of macitentan and the underlying mechanisms, and ii) to test the effectiveness of macitentan as therapeutic agent against MM in xenograft mouse models and in a chick chorioallantoic membrane (CAM) assay.

## Materials and Methods

### Cell Culture

Human MM cell lines RPMI-8226, U266, KMS-12-PE (DSMZ, Braunschneig, Germany), representative of the pattern of expression of ETRs in primary MM cells (8), were cultured as previously described (8). Mycoplasma contaminations were excluded (Mycoplasma Species kit, EuroClone, Milan, Italy). The hypoxia-mimetic agent cobalt chloride (CoCl_2,_ Sigma-Aldrich, Milan, Italy) was used at a concentration of 100 µM. Macitentan was purchased from Selleckchem, Munich, Germany and used at a concentration of 10 µM based on previous titration experiments (9). MM cells were treated for 24 and 48 h with 20 µmol/L of antisense oligonucleotide EZN-2968 (anti-HIF-1α) or its negative control/scrambled EZN-3088 (19, 20) (Enzon Pharmaceuticals Inc. Piscataway, New Jersey).

### Cell Viability and Apoptosis Assay

Cell viability was assessed by MTT (3-(4,5-dimethylthiazol-2-yl)-2,5-diphenyltetrazolium bromide) assay (Sigma Aldrich, Milan, Italy), apoptosis by Annexin-V-FITC (Miltenyi Biotec, Bergisch Gladbach, DE) and Propidium Iodide (Invitrogen, Carlsbad, CA, USA) staining.

### Reverse Transcription Quantitative Real-Time PCR

Total RNA was isolated from MM cell lines using the SV Total RNA Isolation System (Promega, Milan, Italy). Reverse transcription (RT) of 1 µg RNA was performed using the SuperScript III Reverse Transcriptase (Life Technologies, Monza MB, Italy) according to manufacturers’ instructions. The obtained cDNA was amplified by real-time PCR (qPCR) using the Fast SybrGreen MasterMix (Life Technologies, Monza MB, Italy) and intron-spanning primers for ET-1, VEGF-A, Interleukin-8 (IL-8), Adrenomedullin (ADM), and beta-actin (β-ACT). Gene expression was quantified by comparative cycle threshold (Ct) method, by normalizing Ct values to the housekeeping gene β-ACT and calculating relative expression values. The following primers were used: ET-1 forward 5’-CCCTGATGGATAAAGAGTGT-3’ and reverse 5’-TCCAAGTCTAAATCTGTGTCCTG-3’; VEGF-A forward 5’-GAGCCTTGCCTTGCTGCTCTA-3’ and reverse 5’-CACCAGGGTCTCGATTGGATG-3’; IL-8 forward 5’-TACTCCAAACCTTTCCACCC-3’ and reverse 5’-AACTTCTCCACAACCCTCTG-3’; ADM forward 5’-AAGTACTTGGCAGCTCACTCTC-3’ and reverse 5’-CCCACTTATTCCACTTCTTTCG-3’; β-ACT forward 5’-AAAGACCTGTACGCCAACAC-3’ and reverse 5’-GTCATACTCCTGCTTGCTGAT-3’.

### Western Blotting

Following cells lysis, protein cell extracts (40 µg) were separated on 10% SDS-polyacrylamide gel and transferred to a nitrocellulose membrane. Immunoblots were performed overnight using the following primary antibodies diluted 1:1,000: anti-HIF-1α #36169, anti-phospho-ERK1/2 #4370, anti-ERK1/2 #4695, anti-cleaved caspase-3 #9664S and anti-GAPDH #5174S (Cell Signaling Technology, Danvers, USA), anti-VEGF-A #46154 (Abcam, Cambridge, UK). An Ig anti-rabbit #7074S 1:1000 secondary antibody was used (Cell Signaling Technology, Danvers, USA). Blots were then developed using ECL (Merck, Milan, Italy) and images were acquired by Image Quant Las 4000mini (GE Healthcare Life Sciences, Milan, Italy).

### In Vivo Studies

NOD/Shi-scid/IL-2Rγnull (NOG) mice were purchased from Taconic (Germantown, NY, USA). Four groups of five four-to six-week-old female mice were subcutaneously injected with 5 × 10^6^ RPMI 8226 cells. At day +7 post-injection, mice were daily treated with macitentan (30 mg/kg) (21) or vehicle (Methocell 0.05% and TWEEN 80 0,05%) *per os* for four weeks. At the end of treatment mice were sacrificed and tumor masses were measured with a caliper (tumor mass in mm^3^ was calculated using the formula π/6 larger diameter x smaller diameter^2^) and analyzed by immunohistochemistry (IHC). Simultaneously, four groups of five 4–6-week-old mice animals totally irradiated (1.2 Gy,^137^Cesium source) were injected into the tail vein with 2 × 10^6^ U266 cells. At day 7 post-injection, mice were daily treated with macitentan (30 mg/kg) or vehicle (Methocell 0,05% and TWEEN 80 0,05%) *per os.* Following a 7-day treatment, mice were sacrificed, and MM BM burden was evaluated as percentage of viable human CD45^+^CD138^+^ cells over total viable human CD45+ TOPRO3 negative cells. The day of beginning and the length of macitentan treatment were established based on preliminary experiments. RPMI-8226 and U266 cell lines were chosen on the basis of the literature data for subcutaneous and intra-tail injection of MM cell lines, respectively (22).

### Flow Cytometric Analysis

BM cells were flushed from the tibia of adult NOD/Shi-scid/IL-2Rγnull (NOG) mice, red blood cells lysed, and washed twice in PBS. Subsequently, mice BM cells were stained with TOPRO-3 APC, anti-CD45 PE, anti-CD138 V500 (Biolegend, London, UK) and anti-HIF-1α (Cell Signaling Technology, Danvers, USA). Median fluorescence intensity (MFI) was measured by FacsCanto flow cytometer (Becton-Dickinson, New Jersey, USA) and analyzed by 9.3.3 FlowJo software (Tree Star, Ashland, OR, USA).

### Immunohistochemistry and Digital Analysis

After antigen retrieval, sections were incubated with anti-Cleaved Caspase-3 (rabbit polyclonal, diluted 1:600, Trevigen) or with PECAM-1 (goat, clone M-20, diluted 1:600 from Santa Cruz Biotechnology). Reactions were revealed using Goat-on-Rodent-HRP-Polymer (BIOCARE) or EnVision+System HRP Labelled polymer anti-Rabbit (Dako, Agilent, Santa Clara, CA, USA) followed by DAB. Slides were digitalized using ScanScope CS Slide Scanner (Aperio Technologies, Leica, Milan, Italy) at 40 x magnifications and analyzed by Image Scope. PECAM and Caspase-3 analysis were obtained using Positive pixel count v9 (Leica, Milan, Italy).

### Enzyme-Linked Immunosorbent Assay

ET-1, IL-8 and ADM levels were measured in 24 h supernatants from MM cell cultures by ET-1 Quantikine ELISA kit (R&D System, Minneapolis, USA, Canada), IL-8 ELISA (Mabtech, Nacka Strand, Sweden), and ADM Quantikine ELISA kit (R&D System, Minneapolis, USA, Canada). Assay sensitivity was 0.207 pg/mL for ET-1, 4 pg/ml for IL-8, and 7.5 pg/ml for ADM.

### Chorioallantoic Membrane Assay

CAM assay was established as previously described (23). Briefly, alginate beads were prepared by dissolving 6% (w/v) alginic acid sodium salt (Sigma Aldrich) in sterile milliQ H_2_O overnight at 4°C. Then, 2 µL of the solution were added with 2 µL of PBS containing 1.5 x10^4^ RPMI-8226 cells, in the presence of either 0.1% DMSO (vehicle) or macitentan to a final concentration equal to 20 or 40 pmol/pellet. The gelation reaction was obtained by exposing the pellets to 0.1 M CaCl_2_. Alginate beads were then placed on the top of the embryo CAM of fertilized White Leghorn chicken eggs at day 11 of incubation. After 72 h, newly formed microvessels converging towards the implant were counted under a stereomicroscope (STEMI-SR, Zeiss, Germany) at × 5 magnification.

### Statistical Analysis

Mice sample size was chosen as previously described (24). ANOVA test was used to analyze the differences among multiple means. Expression of treatment with respect to controls was analyzed using Student’s t test. Statistical analyses were performed by GraphPad PRISM^®^ version 5-0c.

## Results

### Macitentan Exerts Anti-Proliferative Effects in Multiple Myeloma Cells by Inhibiting the MAPK/ERK and Pro-Survival Pathways

**Figure 1:**
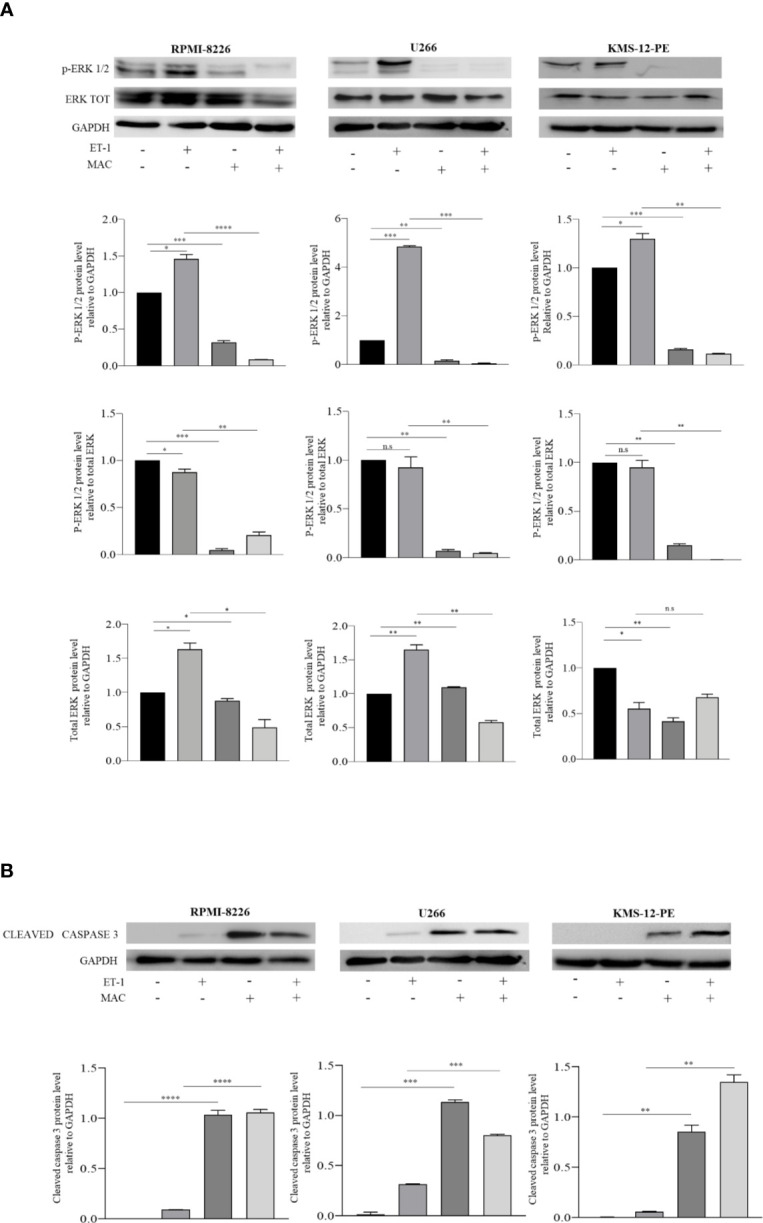
Macitentan modulates basal and ET-1-induced p-erk1/2 phosphorylation and activates caspase 3 cleavage. **(A)** Western blots showing the up-regulation of basal p-ERK following treatment of MM cell lines with ET-1 (100 nM) and the down-modulating effects of macitentan (10 µM) on both basal and ET-1-induced p-ERK over 48 h culture (upper). Densitometric evaluations reporting mean ± SEM (lower). **(B)** Western blots showing the appearance of cleaved form of caspase 3 following treatment of MM cell lines with macitentan (10 µM) over 48 h culture in the presence or absence of ET-1 (100 nM) (upper). Densitometric evaluations reporting mean ± SEM (lower). GAPDH was used as internal control. Each experiment is representative of three replicates. MAC, Macitentan. *p < 0.05, **p <0.005, ***p < 0.001, ****p < 0.0001.

Preliminary results had shown that macitentan exerts significant anti-survival and anti-proliferative activities towards RPMI-8226, U266, and KMS-12-PE cells (9) (**Supplementary Figure 1**), the first two cell lines expressing both ET_A_R and ET_B_R on the membrane, the third one (KMS-12-PE cells) ET_A_R only. To evaluate the mechanisms underlying the anti-proliferative action exerted by macitentan, we examined the MAPK/ERK signaling pathway, which is known to be involved in ET-1-mediated autocrine and paracrine pro-tumorigenic functions (7). As shown by the Western blots in **Figure 1A**, basal levels of p-erk1/2 were found in RPMI-8226, U266, and KMS-12-PE cells under resting conditions. Upon 48 h culture with ET-1, MM cell lines further increased p-erk1/2 expression. By densitometric analysis, macitentan significantly reduced p-erk1/2 levels both in resting and ET-1-stimulated cell lines (**Figure 1A**), thus showing its capacity to antagonize either the autocrine (8) and paracrine activation of ET-1 axis in MM cells. We then observed that the expression of cleaved caspase-3 increased in both resting and ET-1-stimulated cells treated with macitentan, thus better characterizing the pathways downstream of ET-1Rs that may be involved in the resistance to apoptosis (**Figure 1B**). Taken together, our findings confirm the pro-survival activity of the ET-1 axis and its role as therapeutic target in MM. The capacity of macitentan to interfere with major signaling pathways elicited by both autocrine and exogenous ET-1 is also highlighted.

### Macitentan Inhibits Tumor Growth in Multiple Myeloma Xenograft Mouse Models

We next investigated whether macitentan may affect MM cell viability and growth *in vivo*. In order to address this question, we analyzed the effects of treatment with macitentan in NOG mice injected with MM cells either *via* intra-caudal vein or subcutaneously.

**Figure 2:**
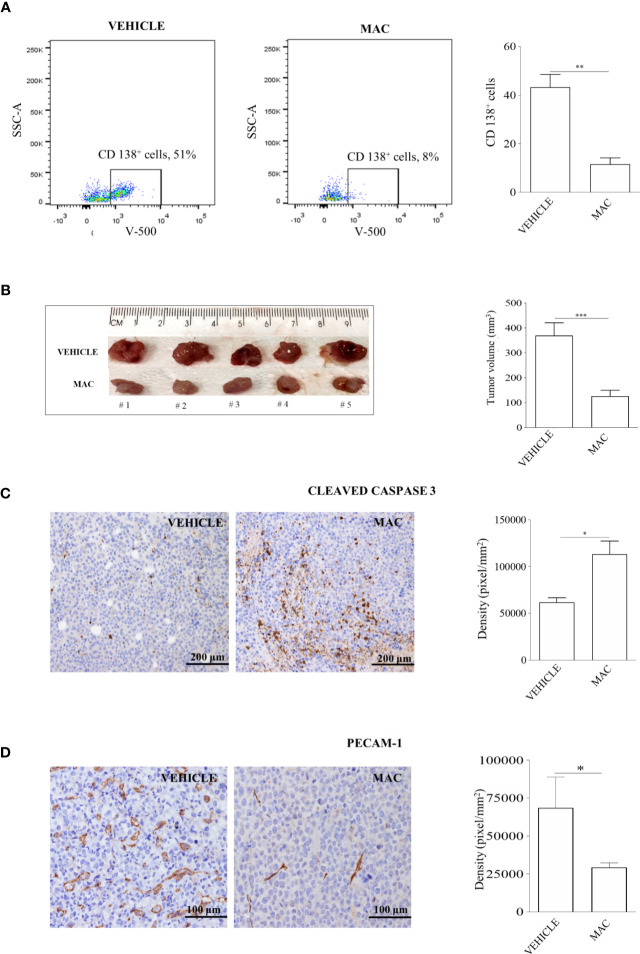
Macitentan inhibits MM cell viability in BM and reduces tumor masses of xenograft mouse models. **(A)** Percentage of viable CD138^+^ cells identified by cytofluorimetric analysis in BM of NOG mice intravenously injected with U266 cells and undergoing treatment with vehicle or macitentan for 7 days (left), and graph representing the significant reduction of viable CD138^+^ cells in the macitentan-treated group (right). **(B)** Representative image of tumor masses from NOG mice subcutaneously injected with RPMI-8226 cells and undergoing 5 weeks treatment with vehicle or macitentan for 5 days/week (left), and graph representing the significant reduction of tumor volumes in the macitentan-treated group (right). **(C)** Immunostaining of cleaved caspase 3 performed on formalin fixed paraffin-embedded samples (n=12) obtained from tumor masses of mice treated with vehicle or macitentan (left), and graph representing the significant increase of cleaved caspase 3 in tumor masses from macitentan-treated animals. **(D)** Immunostaining of PECAM-1 performed on formalin fixed paraffin-embedded samples (n=12) obtained from tumor masses of mice treated with vehicle or macitentan (left), and graph representing the significant reduction of PECAM-1 staining in macitentan-treated animals. Original magnification x40. *p < 0.05, **p < 0.005, ***p < 0.001.

A first group of 20 mice was injected intra-venously with 2x10^6^ U266 cells. Starting from the engraftment (day +7 from injection) and for the subsequent 7 days, mice were treated orally either with macitentan at a dosage of 30 mg/kg (n=10) or with vehicle only (n=10), and then sacrificed. Notably, following injection of U266 cells, control mice displayed hair alterations, weight loss and limbs paralysis as for disease progression (not shown). BM cells, collected by tibial flushing from macitentan-treated and untreated mice, were then analyzed for viability by flow cytometry after anti-CD45 and anti-CD138 staining (**Supplementary Figure 2**). The viable (i.e., TOPRO-3 negative) CD138^+^ cell fraction was significantly reduced in BM samples from macitentan-treated mice as compared to controls (11 *vs* 44%, *p*=0.001) (**Figure 2A**), thus showing that macitentan can reach effective therapeutic concentrations in the tissue primarily affected by MM cell expansion.

A second group of 20 mice was injected subcutaneously with 5x10^6^ RPMI-8226 cells and monitored for subcutaneous tumor mass development. Starting from day +7 after injection, mice were treated with a macitentan daily oral dose of 30 mg/kg, 5 days a week, for 4 weeks (n=10) or with vehicle only (n=10), and then sacrificed. At the end of treatment all animals developed well-delimited tumors in the site of injection that were removed and measured. As shown in **Figure 2B**, tumor masses from macitentan-treated mice had a median size significantly reduced as compared to controls (125 mm^3^ *vs* 370 mm^3^, *p*=0.003). Consistently with previous *in vitro* findings, higher expression of cleaved caspase-3 was observed in histological specimens from macitentan-treated mice as compared to controls (**Figure 2C**). Interestingly, PECAM1 staining revealed a significantly diminished microvascular density (MVD) in RPMI-8226 tumor masses from macitentan-treated mice, as compared to controls (**Figure 2D**), thus suggesting that in MM macitentan could also exert an anti-angiogenic activity.

Overall, our *in vivo* findings extend previous *in vitro* results regarding the anti-proliferative effects of ET-1Rs blockade in MM cells and support the role for macitentan as an effective therapeutic agent against MM. In addition, the significantly reduced MVD observed in tumors from treated mice as compared to controls anticipates a possible anti-angiogenic activity of macitentan in MM.

### Macitentan Downregulates Hypoxia Inducible Factor-1alpha, a Crucial Mediator of Multiple Myeloma Angiogenesis

**Figure 3:**
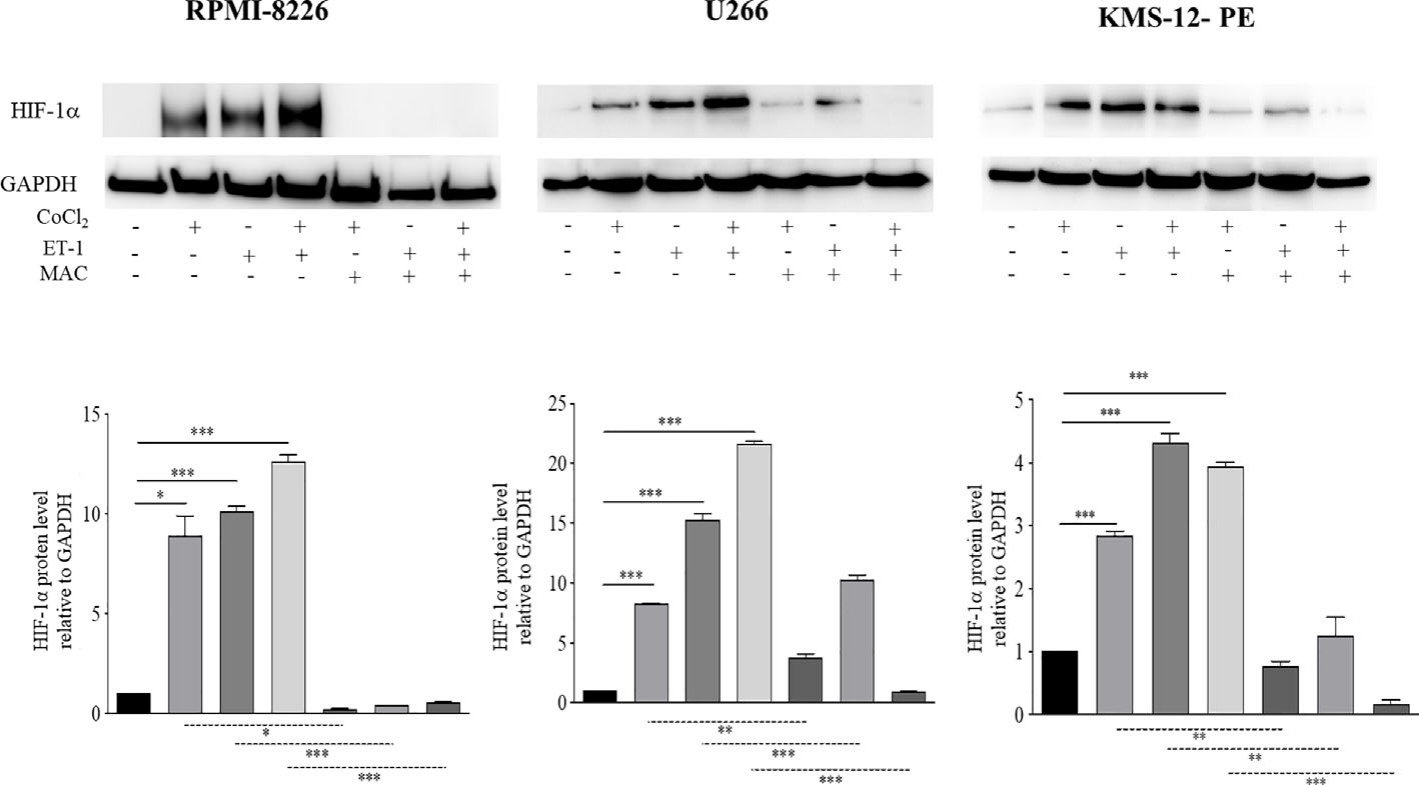
Macitentan significantly reduces ET-1-induced HIF-1α expression. Western blots showing the effects of macitentan (10 µM) on up-regulation of HIF-1α in MM cell lines exposed to the hypoxia-mimetic agent CoCl_2_ (100 µM), to ET-1 (100 nM), and to CoCl_2_ plus ET-1 for 48 h (upper). Densitometric evaluations reporting mean ± SEM (lower). GAPDH was used as internal control (lower). Each experiment is representative of three replicates. *p < 0.05, **p < 0.005, ***p < 0.001.

HIF-1α plays a crucial role in the adaptive responses to hypoxia of tumor cells through transcriptional activation of downstream genes required for tumor survival and progression, including those for angiogenesis (25). It is known that MM cells up-regulate HIF-1α in the hypoxic BM niche, which in turn promotes MM progression (26). Previous studies demonstrated the capacity of ET-1 to induce HIF-1α in solid tumors (12–14). In addition recent network-based analysis highlighted the significant ET-1 gene enrichment in the HIF-1α signaling pathway of PCs from BM samples of patients affected by MM and MGUS (18). Consequently, we tried to assess whether ET-1 was capable of up-regulating HIF-1α in MM, similarly to hypoxia. We therefore evaluated HIF-1α protein levels in RPMI-8226, U266, and KMS-12-PE cells cultured for 48 h in the presence of the hypoxia-mimetic agent CoCl_2_, ET-1, or their combination. As shown in **Figure 3** both CoCl_2_ and ET-1 as well as their combination induced HIF-1α in all cell lines. Interestingly, ET-1 induced HIF-1α similarly to CoCl_2_ in RPMI-8226 and, at higher levels, in U266 and KMS-12-PE cells (*p*<0,001). The combination of ET-1 plus CoCl_2_ promoted a further induction of HIF-1α in RPMI-8226 and U266 cells. Importantly, macitentan significantly inhibited both CoCl_2_- and ET-1-induced HIF-1α, also reducing the combined effect of CoCl_2_ plus ET-1 (**Figure 3**). Taken together these results demonstrate that, in MM, the ET-1 axis activates and potentiates HIF-1α expression, which is significantly down-regulated by macitentan. Consistently with these results, we observed by flow cytometry that viable (i.e., TOPRO3 negative) U266 cells isolated from BM of macitentan-treated mice displayed a lower HIF-1α median fluorescence intensity (MFI) than controls (219 *vs* 12, *p*<0,001) (**Supplementary Figure 3**).

### Macitentan Disrupts the Mutual Dependence Between ET-1 Axis and Hypoxia Inducible Factor-1alpha

**Figure 4:**
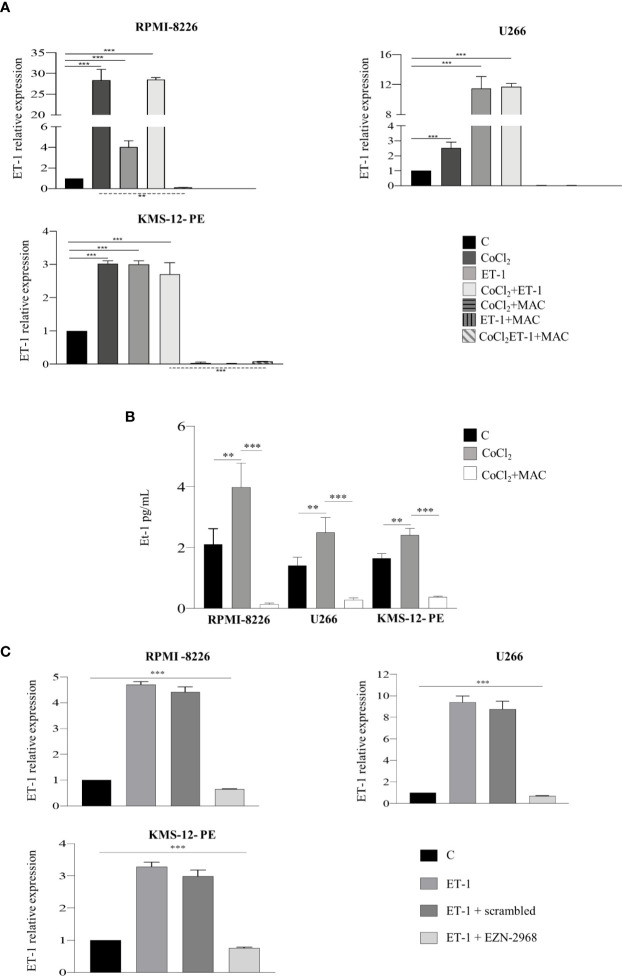
ET-1 expression and release are HIF-1α-dependent. **(A)** RT-qPCR analysis showing ET-1 mRNA expression in MM cell lines treated with CoCl_2_ (100 µM), ET-1 (100 nM) and CoCl_2_ plus ET-1 alone or in combination with macitentan (10 µM) for 3 h, normalized on β-actin mRNA. Data represent the mean value ± SEM. **(B)** ELISA measuring ET-1 levels in 48 h supernatants from MM cell lines treated with CoCl_2_ alone or in combination with macitentan. Data represent mean value ± SEM. **(C)** RT-qPCR analysis showing ET-1 mRNA expression in MM cell lines HIF-1α-silenced with the antisense oligonucleotide EZN-2968 or scrambled oligonucleotide (20 µmol/L) for 48 h, and exposed to ET-1 100 nM. ET-1 mRNA expression was normalized on β-actin. Each experiment is representative of three replicates. **p < 0.005, ***p < 0.001.

Our previous work had shown that both MM and BM microenvironment cells, including ECs, express and release ET-1, therefore allowing us to demonstrate an autocrine and paracrine pro-tumor activity of the ET-1 axis in MM (8). As ET-1 gene transcription is dependent on HIF-1α, we hypothesized that MM cells could increase the transcription and the release of ET-1 in the hypoxic BM niche. To evaluate whether MM cells are capable of expressing and releasing higher levels of ET-1 in hypoxic conditions, thus self-sustaining the pro-tumor activity of the ET-1 axis, we cultured RPMI-8226, U266 and KMS-12-PE cells in the presence of CoCl_2_. As reported in **Figures 4A, B**, upon exposure to CoCl_2_ MM cells increased significantly ET-1 gene transcription and release, which were reduced by the addition of macitentan. Interestingly, ET-1 gene transcription increased upon culture of MM cells with ET-1 itself and by its combination with CoCl_2_, while being significantly down-regulated by macitentan in all experimental settings (**Figure 4A**). Finally, upon exposure to ET-1, MM cells HIF-1α- silenced (**Supplementary Figures 4A, B**) displayed a significantly reduced ET-1 gene transcription (**Figure 4C**), thus indicating that the regulation of ET-1 expression is mediated by HIF-1α.

Overall, these findings indicate that ET-1 axis and HIF-1α pathway in MM are mutually dependent in an autocrine and paracrine fashion, and that macitentan may target crucial HIF-1α-dependent pathways.

### Macitentan Exerts a Significant Antiangiogenic Activity by Downmodulating Cytokine Expression and Release by Multiple Myeloma Cells

**Figure 5:**
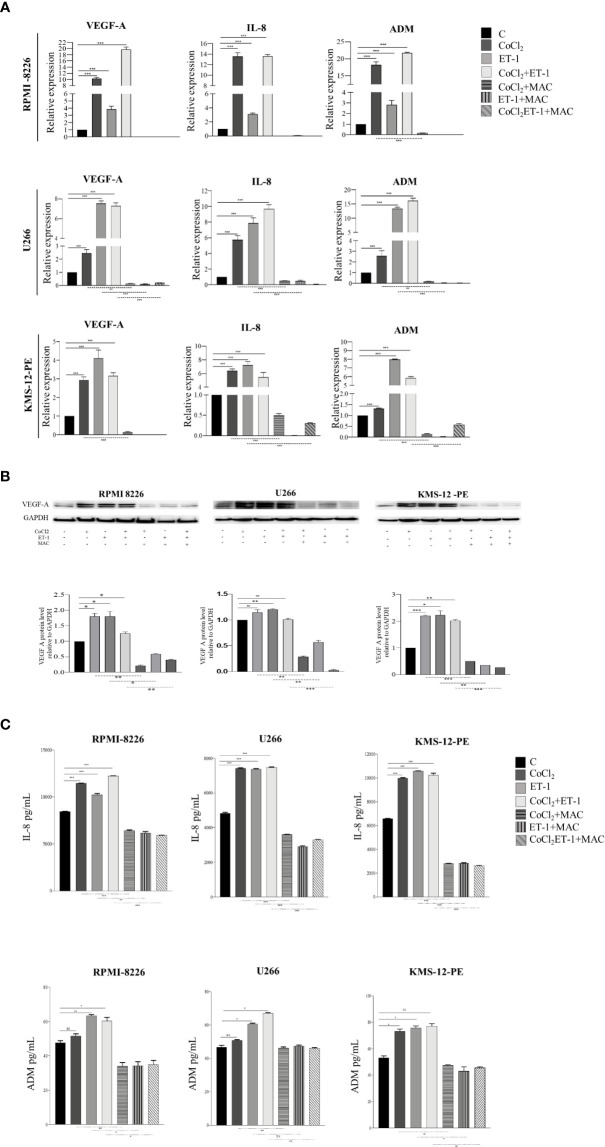
Macitentan inhibits pro-angiogenic cytokine gene transcription and protein production/release. **(A)** RT-qPCR analysis of VEGF-A, IL-8 and ADM mRNA expression in MM cell lines treated with CoCl_2_ (100 µM), ET-1 (100 nM) and CoCl_2_ plus ET-1 in the absence or presence of macitentan (10 µM) for 3 h, normalized on β-actin mRNA. Data represent the mean value ± SEM. **(B)** Western blot showing the modulation of VEGF-A in MM cell lines stimulated with CoCl_2_, ET-1, and CoCl_2_ plus ET-1 in the absence or presence of macitentan (10 µM) for 48 h (upper). GAPDH was used as internal control. Each experiment is representative of three replicates. Densitometric evaluations reporting mean ± SEM (lower). **(C)** IL-8 and ADM levels measured by ELISA in supernatants from MM cell lines stimulated with CoCl_2_ (100 µM), ET-1 (100 nM), and CoCl_2_ plus ET-1 in the absence or presence of macitentan (10 µM) for 48 h. Data represent mean value ± SEM. Each experiment is representative of three replicates. *p < 0.05, **p < 0.005, ***p < 0.001.

Angiogenic switch represents a crucial step towards MM progression (27). Several experimental evidences have already shown that, in MM cells, hypoxic condition targets the transcription of a number of pro-angiogenic cytokines genes *via* HIF-1α, including VEGF-A, IL-8, and ADM (28, 29). As ET-1 up-regulates HIF-1α, we evaluated whether the transcription of HIF-1α-dependent pro-angiogenic genes in MM may be mediated by the ET-1 axis. We thus evaluated by RT-qPCR the transcription of VEGF-A, IL-8 and ADM in RPMI-8226, U266 and KMS-12-PE cells exposed for 3 h to CoCl_2_, ET-1, or their combination. ET-1, either alone or in combination with CoCl_2_, up-regulated in all cell lines the expression of VEGF-A, IL-8 and ADM genes, which in turn were effectively down-modulated by macitentan (**Figure 5**). The capacity of ET-1 to increase VEGF-A, IL-8 and ADM gene transcription was significantly reduced in HIF-1α-silenced MM cells (**Supplementary Figure 5**), further reinforcing the existence of an interplay among ET-1 axis, hypoxia and angiogenesis in MM. The pro-angiogenic activity of the ET-1 axis in MM was then evaluated by measuring VEGF-A, IL-8 and ADM protein levels in RPMI-8226, U266, and KMS-12-PE cells cultured in the presence of CoCl_2_, ET-1, or their combination. As shown by Western blots in **Figure 5B**, VEGF-A, constitutively expressed at low levels by all cell lines, was significantly up-regulated by either CoCl_2_ or ET-1, alone or in combination. In all experimental settings, macitentan down-regulated the expression of VEGF-A. As demonstrated by ELISA analysis, the supernatants from RPMI-8226, U266 and KMS-12-PE cells cultured with CoCl_2_, ET-1, or their combination, contained increased concentrations of IL-8 and ADM, which in turn were significantly reduced by the addition of macitentan (**Figure 5C**). Overall, these findings indicate a crucial role of the ET-1 axis as a mediator of angiogenesis in MM. More importantly, our data highlight the capacity of macitentan to interfere with the expression and release of pro-angiogenic cytokines, such as ET-1 itself, VEGF-A, IL-8 and ADM through the down-modulation of HIF-1α in MM cells.

**Figure 6:**
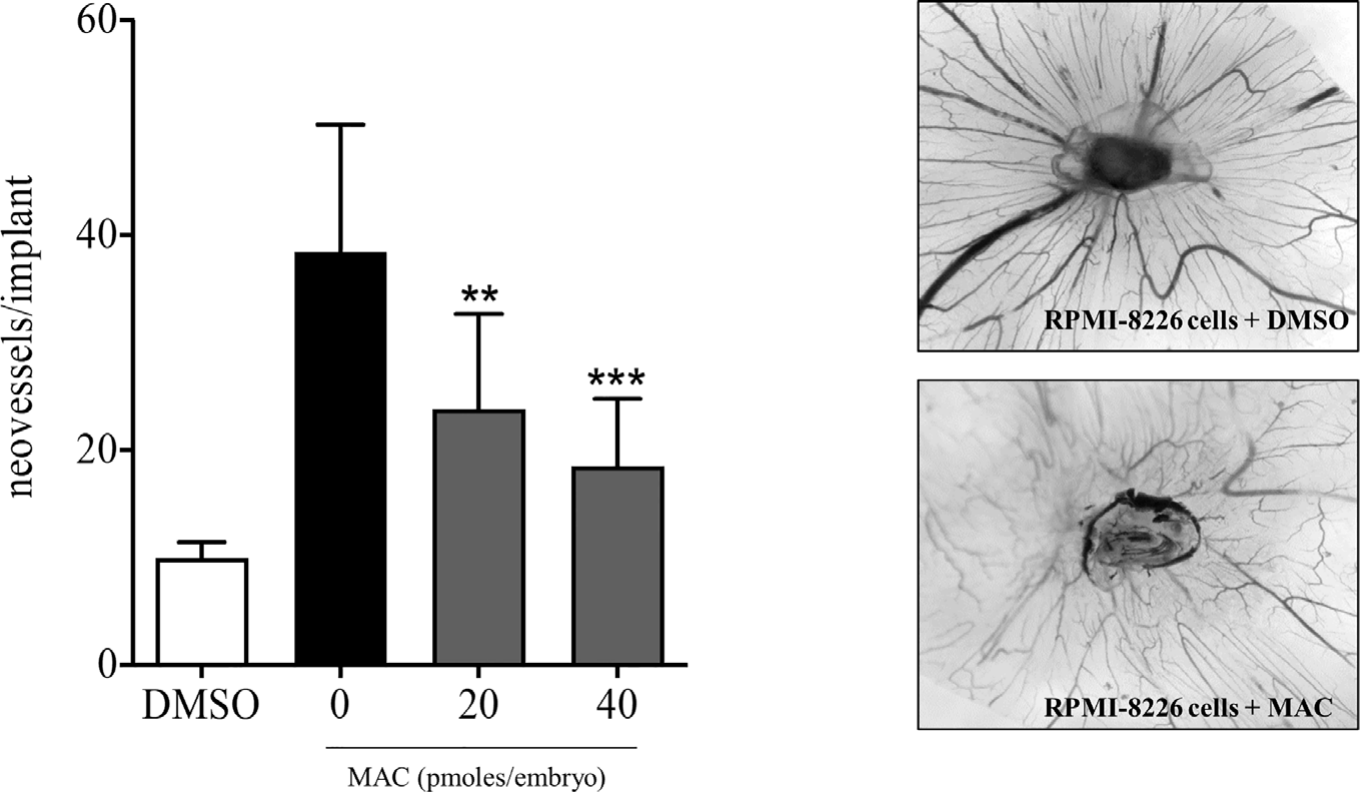
Anti-angiogenic activity of macitentan in CAM assay. Left, graphic representation of the number of newly formed vessels counted at day 14 in chick embryo CAMs implanted at day 11 with alginate beads containing DMSO only or 1.5 x10^4^ RPMI-8226 cells plus DMSO or 1.5 x10^4^ RPMI-8226 cells plus increasing amounts of macitentan. Data are the mean ± SEM of 11-14 eggs per experimental point in two independent experiments. **p < 0.01; ***p < 0.001 *versus* untreated (ANOVA). Right, representative images of CAMs grafted with RPMI-8226 cells in the absence (upper panel) or in the presence (lower panel) of 40 pmol of macitentan.

To demonstrate that macitentan exerts a direct anti-angiogenic activity in MM *in vivo*, we subsequently set-up a chick embrio CAM assay using RPMI-8226 cells, which express basal levels of VEGF-A, IL-8 and ADM, other than ET-1 (**Figures 4B**, **5B, C**). RPMI-8226 cells were therefore grafted in an alginate pellet and the number of newly formed vessels converging towards them was counted. As shown in **Figure 6**, when grafted on the top of the chick embryo CAM, RPMI-8226 cells induced a potent angiogenic response, which was significantly inhibited by the presence of increasing concentrations of macitentan, clearly unveiling its anti-angiogenic activity in MM.

## Discussion

In this study we show that the ET-1 axis increases MM cell growth and angiogenesis both in an autocrine and paracrine manner. Of translational relevance, we demonstrate that in xenograft mouse models macitentan impairs MM cell growth and decreases MM-related vascularity. In our opinion, these data reinforce and extend the role of ET-1 axis as therapeutic target in MM (8, 9, 30).

Our *in vitro* experiments have shown that MM cells express and release increased levels of ET-1 when cultured with CoCl_2_, the latter mimicking the hypoxic conditions of the BM niche. Similar to CoCl_2_, exogenous ET-1 increased HIF-1α levels in MM cells by up-regulating the transcription of HIF-1α-dependent pro-angiogenic genes such as VEGF-A, IL-8, ADM, and ET-1 itself. Under the same experimental settings, macitentan effectively down-modulated HIF-1α, the expression of HIF-1α-dependent pro-angiogenic genes, and the production/release of the corresponding cytokines. These findings appear of particular interest, as HIF-1α may increase angiogenesis in MM BM under the combined influence of hypoxia and ET-1. In agreement with such hypothesis, HIF1-α-silenced MM cells failed to express VEGF-A, IL-8 and ADM, other than ET-1 itself, upon exposure to exogenous ET-1. Significant anti-proliferative effects in terms of MM cell viability in BM and subcutaneous tumor growth were observed *in vivo* in MM mouse models treated with macitentan. In the same models, residual viable MM cells in BM showed a reduced expression of HIF-1α, while subcutaneous masses displayed a significantly lower MVD than controls. In line with these findings, macitentan effectively impaired the induction of angiogenesis by MM cells in CAM assay. Overall, the set of experimental evidence here provided underscores a particular sensitivity of MM cells towards ET-1Rs blockade, which can hamper ET-1-triggered crucial MM pro-survival and pro-angiogenic signaling pathways, such as those involving MAPK/ERK (31) and HIF-1α (28, 32), respectively.

**Figure 7:**
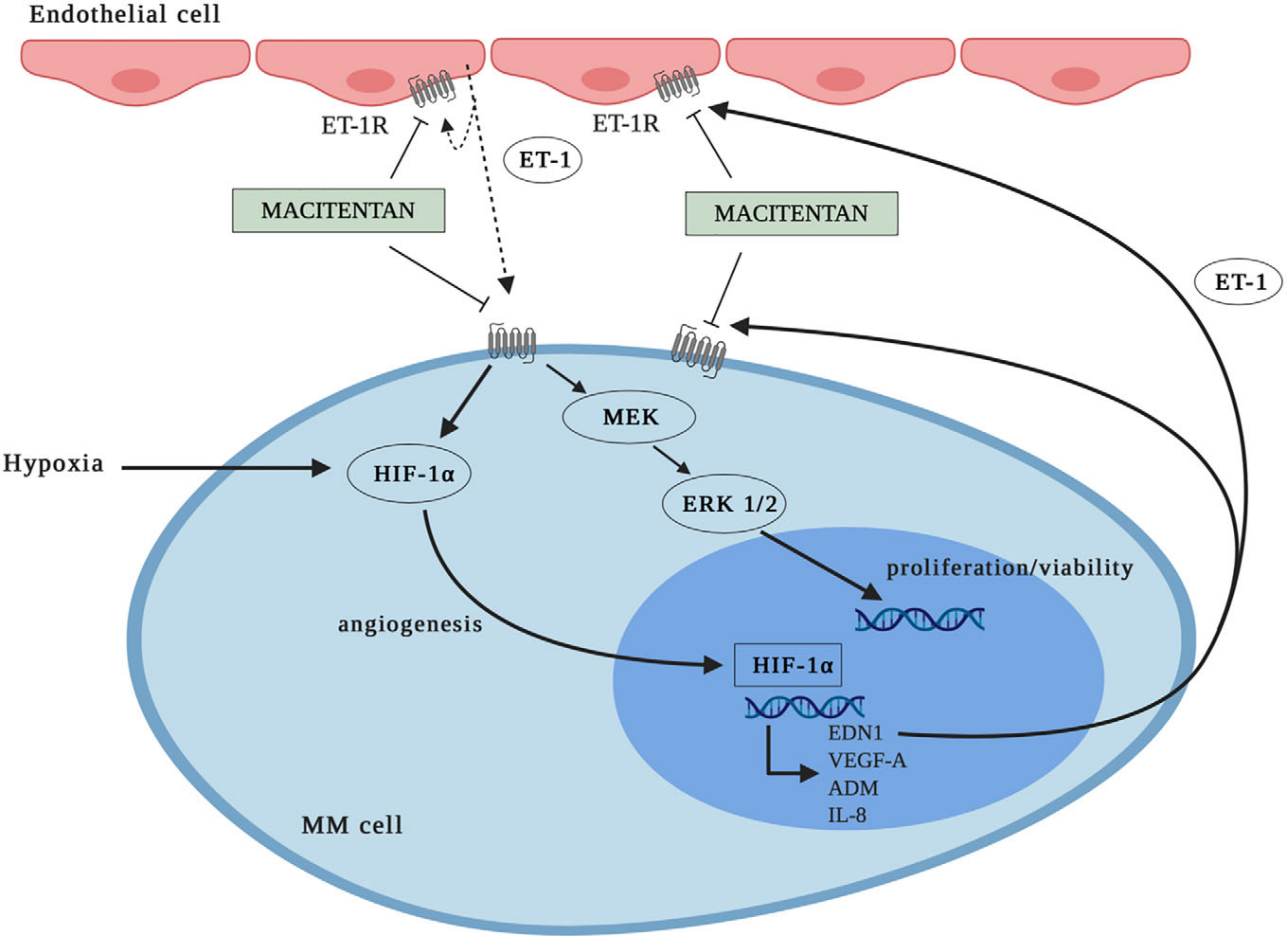
Graphic representation: mechanisms whereby macitentan inhibits MM cell growth and angiogenesis. MM cells express ET_A_R or ET_A_R and ET_B_R. Blockade of ET-1Rs by macitentan targets not only MM cell proliferation/viability (through ERK1/2 down-modulation), but also MM cell angiogenic activity (through HIF-1α down-modulation). According to data in literature, macitentan could also target ET-1 axis in endothelial cells (ECs) through ET_B_R antagonism.

MM is known to interplay through mutual pro-survival/proliferative signals with the BM microenvironment (4, 33), the latter being characterized by angiogenesis as a hallmark of disease progression. Novel drugs targeting not only tumor cells, but also the BM microenvironment have been proved to be highly effective in MM (31). Under this perspective, oral immunomodulatory imide drugs, including thalidomide, lenalidomide, pomalidomide as well as proteasome inhibitors, such as bortezomib, are currently used in clinical protocols due to their anti-proliferative and anti-angiogenic proprieties (27). Our experimental evidence together with literature data indicate that macitentan may also target both tumor and BM microenvironment, as ET-1 axis drives autocrine and paracrine pro-survival and pro-angiogenic signals in both malignant PCs and ECs. Indeed, not only MM cells (8), but also ECs (10) release increased levels of ET-1 through a HIF-1α-dependent activation (34), further reinforcing the hypothesis of an ET-1 axis-mediated mutual support between MM cells and ECs in the hypoxic BM microenvironment. Furthermore, based on ET_A_R and ET_B_R expression by MM cells (8) and ET_B_R by ECs (10), ET-1Rs blockade could target not only MM cells, but also the formation of new vessels, therefore exerting a potent therapeutic action against both MM and angiogenesis. Worthy of note, the expression of HIF-1α by ECs in BM has been also pointed as a therapeutic target in MM (35). At the same time, ET-1Rs blockade could inhibit the autocrine activation of ET-1-axis in both MM cells and ECs (10, 35, 36), thus interrupting an autocrine other than a paracrine pathogenic loop. Of interest, due to its pharmacokinetic properties (10), macitentan can be effective towards MM cells expressing ET_A_R only or ET_A_R and ET_B_R, as well as towards ET_B_R positive ECs (**Figure 7**).

ET-1 axis has been considered for a long time a possible therapeutic target in a number of solid tumors, though with fluctuating results (16). As previously stated, macitentan is a second-generation dual receptor antagonist that has been approved since 2013 for the therapy of PAH in the United States and Europe (37, 38). Macitentan does not appear to require dosage adjustment in patients with hepatic and renal impairment (37), thus presenting a toxicity profile potentially suitable for the treatment of MM, a disease often associated with renal failure. Additionally, macitentan was shown to sensitize tumor cells to different cytotoxic and targeted agents in various preclinical tumor models, including colorectal cancer, glioblastoma, breast, and lung brain metastasis (39). In this regard, our preliminary *in vitro* findings indicated that macitentan can be administered in combination with other drugs already used for MM treatment. Accordingly, we found that macitentan exerts a synergistic action towards MM cells with the proteasome inhibitor bortezomib (9) and with the oral immunomodulatory imide drug pomalidomide (R.A. and C.T. unpublished results).

Overall, our data point out a potential therapeutic role for macitentant in MM patients that deserves to be exploited in further preclinical and phase 1–2 clinical studies, either alone or in combination with other anti-MM drugs.

## Data Availability Statement

The original contributions presented in the study are included in the article/**Supplementary Material**; further inquiries can be directed to the corresponding authors.

## Ethics Statement

The animal study was reviewed and approved by Technical and Scientific Committee of University of Verona and Italian Ministry of Health.

## Author Contributions

RA, BA and TC conceived and/or designed the work that led to submission. RA, DCG, TN, MBu, MBe, BE, BR, TC, VW, GA, PM, and CMA developed the methodology. RA, DCG, TN, MBu, MBe, and LL performed the experiments and/or analyzed the data. RA, BA, GM, PM, GA, CMA, KM, and TC wrote and/or revised the paper. BE, BR, GM, TC, VW, PM, CMA, KM, and TC provided administrative, technical, or material support. BA and TC supervised the study. All authors contributed to the article and approved the submitted version.

## Funding

This work was supported by: The Alessandro Moretti Foundation, Verona, Italy, and the Lions Club Dante Alighieri, Verona, Italy.

## Conflict of Interest

The authors declare that the research was conducted in the absence of any commercial or financial relationships that could be construed as a potential conflict of interest.
