# Supplementary material for: Targeting the Endothelin-1 Receptors Curtails Tumor Growth and Angiogenesis in Multiple Myeloma
Source: Front Oncol. 2021 Jan 8;10:600025. doi: 10.3389/fonc.2020.600025 (PMC7820698; doi:10.3389/fonc.2020.600025)
Supplement: Supplementary file 1 [file DataSheet_1.docx]

**FIGURE S1.** *Annexin V-FITC/PI staining of MM cell lines*. Cells were treated with macitentan 10 μM for 48 hours. Annexin V-FITC single-positive cells were early apoptotic cells. Annexin V-FITC and PI double-positive cells were necrotic or late apoptotic cells, and PI single-positive cells were nude nuclear cells. MAC: Macitentan.

**FIGURE S2.** *Representative gating strategy to identify viable, CD138+ U266 cells in mouse BM*. Total cells were gated on a forward (FSC)/side (SSC) scatter plot (A). Following doublets exclusion (B), viable TOPRO-3 negative cells were selected (C). After CD45+ cell gating (D), U266 cells were identified using CD138 antibody (E).

**FIGURE S3.** *Down-regulation of HIF-1α MFI in MM cells from BM of macitentan-treated mice*. Graph representing HIF-1α median fluorescence intensity (MFI) in viable CD45^+^/CD138^+^ cells identified by flow cytometry analysis in BM from NOG mice intravenously injected with U266 cells and undergoing treatment with vehicle or macitentan (30 mg/kg) for 7 days. MAC: Macitentan. *** *p <*0.001.

**FIGURE S4.** *Evidence of HIF-1α silencing by EZN 2968 oligonucleotide*. **(A)** RT-qPCR analysis of HIF-1α mRNA expression in MM cell lines treated with the anti-HIF-1α oligonucleotide EZN-2968 or EZN-3088 scrambled oligonucleotide (20 μmol/L) for 24 hours (left) and 48 hours (right), and normalized on β-actin mRNA, **(B)** TOPRO-3 analysis of viability of MM cell lines treated with oligonucleotides EZN-2968 and EZN-3088 (20 μmol/L) for 24 hours (left) and 48 hours (right). Each graph represents the mean ± SEM of three independent experiments. ** *p* < 0.001 *vs* control.

**FIGURE S5.** *HIF-1α silencing reduces ET-1-induced transcription of pro-angiogenic genes in MM cells.* RT-qPCR analysis of VEGF-A, IL-8 and ADM mRNA expression in MM cell lines treated with the anti-HIF-1α oligonucleotide EZN-2968 or scrambled oligonucleotide (20 mmol/L) for 24 and 48 hours, normalized on β-actin mRNA. The graph represents mean ± SEM of three independent experiments. VEGF-A: Vascular endothelial growth factor A, IL-8: Interleukin-8, ADM: Adrenomedullin. * *p* < 0.05, ** *p* <0.005, *** *p* < 0.001.
