# Supplementary figures and images for: Targeting the Endothelin-1 Receptors Curtails Tumor Growth and Angiogenesis in Multiple Myeloma
Source: Front Oncol. 2021 Jan 8;10:600025. doi: 10.3389/fonc.2020.600025 (PMC7820698; doi:10.3389/fonc.2020.600025)

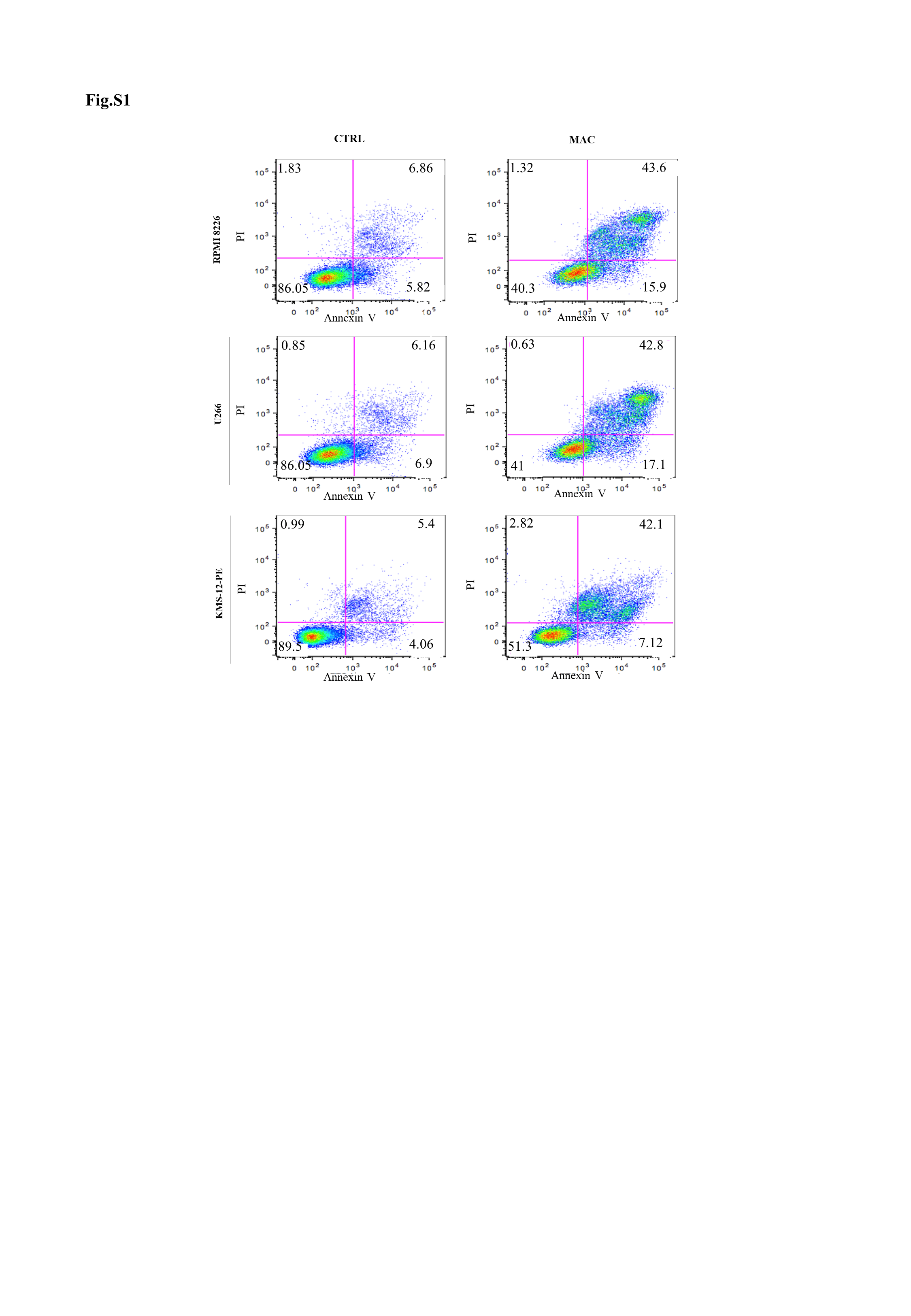

Supplement: Supplementary file 2 [file Image_1.tif]

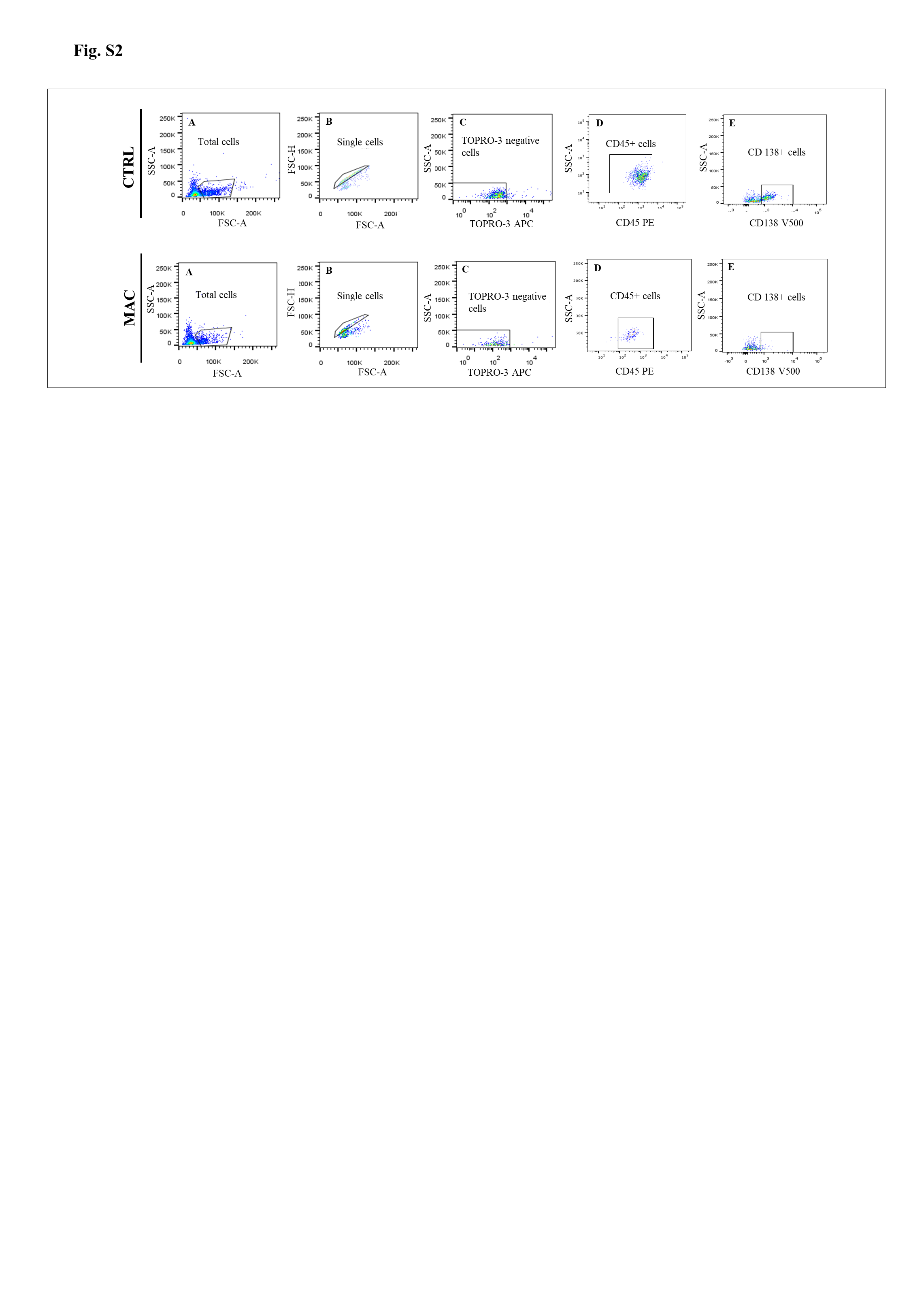

Supplement: Supplementary file 3 [file Image_2.tif]

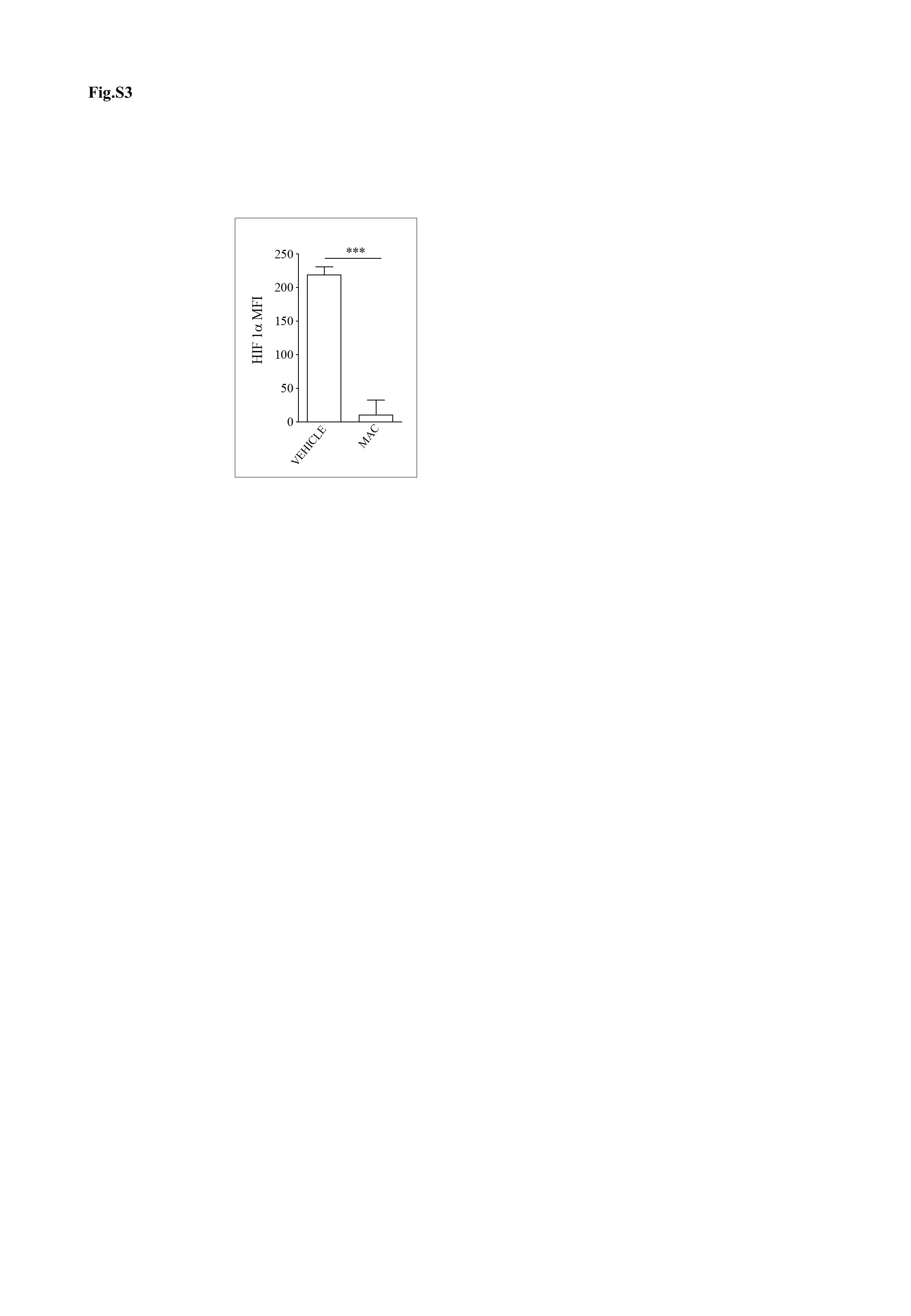

Supplement: Supplementary file 4 [file Image_3.tif]

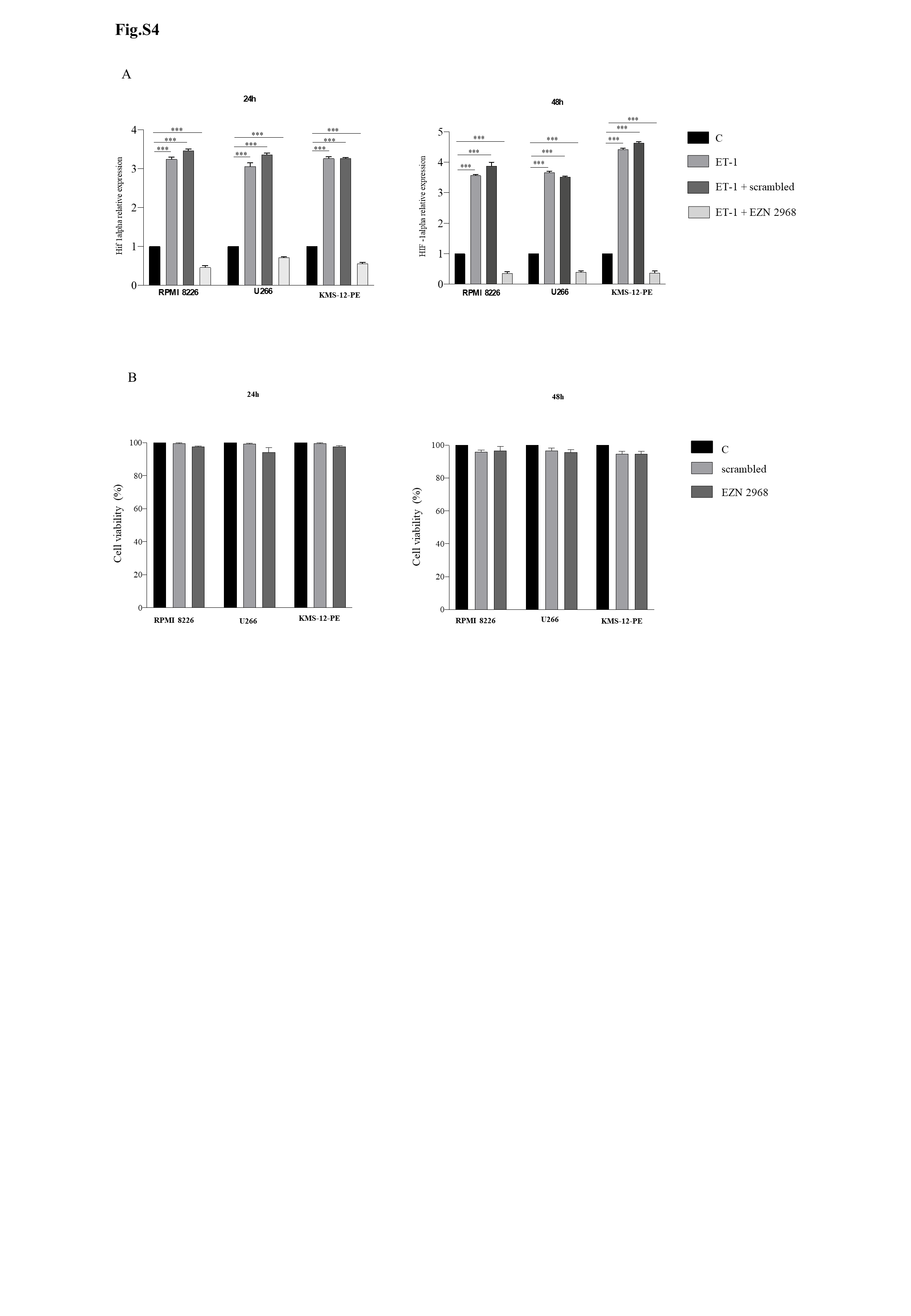

Supplement: Supplementary file 5 [file Image_4.tif]

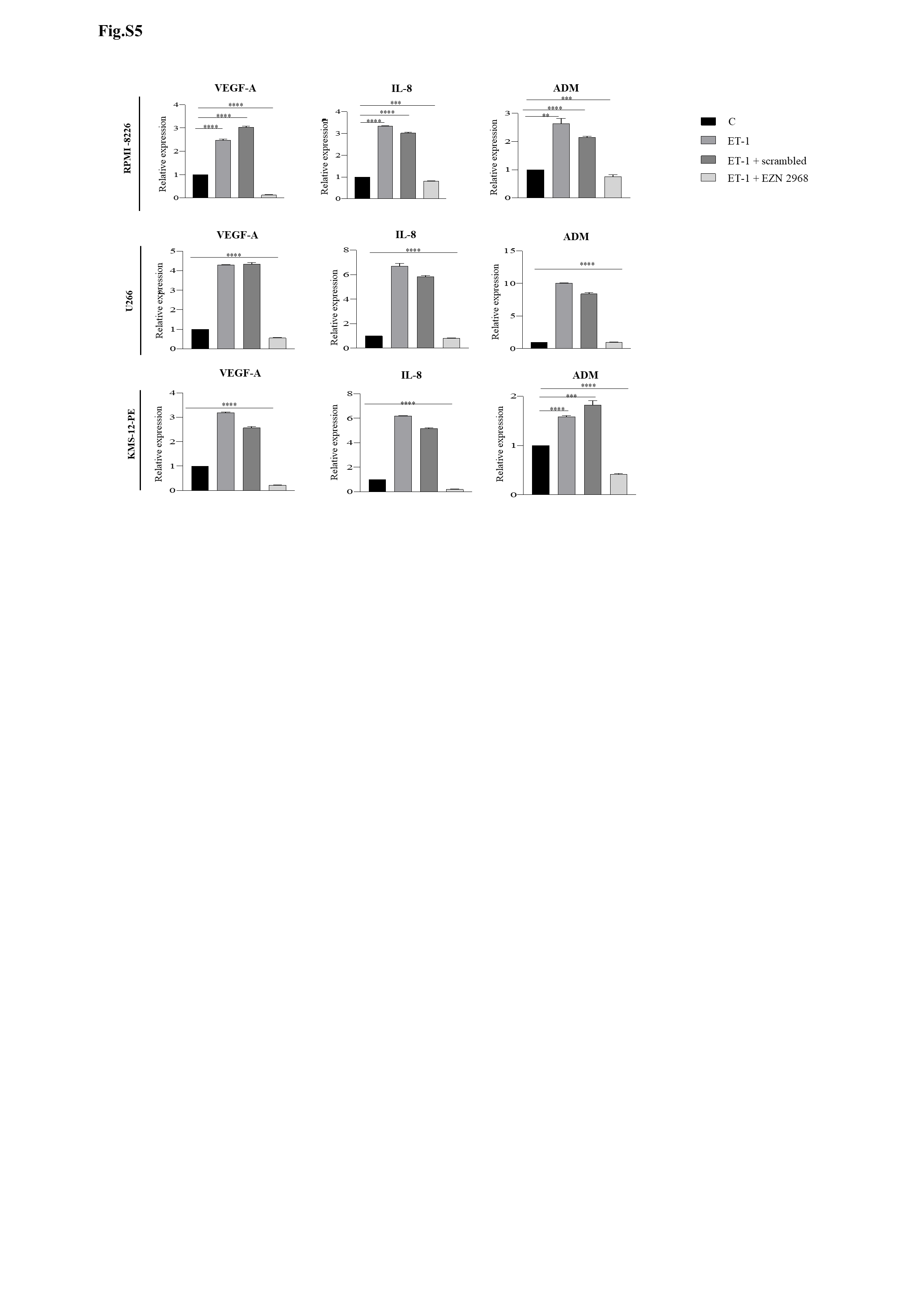

Supplement: Supplementary file 6 [file Image_5.tif]
